# A preliminary study of comprehensive behavioral intervention for tics in Chinese children with chronic tic disorder or Tourette syndrome

**DOI:** 10.3389/fpsyt.2022.997174

**Published:** 2022-11-11

**Authors:** Wen Xu, Qiang Ding, Ying Zhao, Wenqing Jiang, Jingjing Han, Jinhua Sun

**Affiliations:** ^1^Department of Psychological Medicine, Children’s Hospital of Fudan University, Shanghai, China; ^2^Shanghai Mental Health Center, Shanghai Jiao Tong University, Shanghai, China; ^3^School of Medicine, Tongji University, Shanghai, China

**Keywords:** Tourette syndrome, comprehensive behavioral intervention for tics (CBIT), chronic tic disorders (CTD), pharmacological treatment, life quality

## Abstract

**Objective:**

To investigate the adaptability of Comprehensive Behavioral Intervention for Tics (CBIT) for a Chinese population, and evaluate the efficacy of combined CBIT and pharmacotherapy (CBIT + PT) compared to CBIT or pharmacotherapy (PT) alone for reducing tics and for improving the quality of life (QoL) in a sample of Chinese children with chronic tic disorders (CTD) and Tourette syndrome (TS).

**Materials and methods:**

In this 10-week randomized controlled pilot trial, 37 outpatients aged between 6 and 16 years affected by TS and CTD were randomly assigned to receive CBIT (*n* = 22) or PT alone (*n* = 15). Considering the feasibility, the patients allocated to the CBIT treatment group could further choose whether to simultaneously take medicine voluntarily, resulting in a CBIT alone group (*n* = 12) and a CBIT + PT group (*n* = 10).

**Results:**

At baseline, no significant difference was found between the three groups in the demographic and clinical characteristics (*p* > 0.05). All three groups showed a significant reduction in tic severity after treatment assessed by the Yale Global Tic Severity Scale (YGTSS) severity score [*F*_(2_,_33)_ = 35.05, *p* < 0.001, η*_*p*_*^2^ = 0.51], the score of the Clinical Global Impression scale for Improvement (CGI-I) [*F*_(1_,_34)_ = 13.87, *p* = 0.001, η*_*p*_*^2^ = 0.29], and YGTSS impairment score [*F*_(2_,_33)_ = 31.71, *p* < 0.001, η*_*p*_*^2^ = 0.48]. Significant interactions were found between the time-point and group in emotional functioning [*F*_(2_,_29)_ = 4.39, *p* = 0.02, η*_*p*_*^2^ = 0.23], psychosocial functioning [*F*_(2_,_29)_ = 5.93, *p* = 0.007, η*_*p*_*^2^ = 0.29], and total QoL score [*F*_(1_,_34)_ = 3.72, *p* = 0.04, η*_*p*_*^2^ = 0.20] of Pediatric Quality of Life Inventory (PedsQL 4.0) for children suggesting a significantly larger improvement in emotional functioning, psychosocial functioning, and total QoL score of the life quality in the CBIT group for children self-report. PedsQL for proxy report only showed a significant main effect of time-point in physical functioning [*F*_(1_,_33)_ = 8.35, *p* = 0.01, η*_*p*_*^2^ = 0.2], emotional functioning [*F*_(1_,_33)_ = 10.75, *p* = 0.002, η*_*p*_*^2^ = 0.25], psychosocial functioning [*F*_(1_,_34)_ = 11.38, *p* = 0.002, η*_*p*_*^2^ = 0.26], and total Qol score [*F*_(1_,_34)_ = 13.21, *p* = 0.001, η*_*p*_*^2^ = 0.29].

**Conclusion:**

CBIT is probably effective in reducing tic severity in Chinese children with tic disorders. CBIT + PT may not be superior to CBIT alone in reducing tic severity and improving quality of life. CBIT alone showed advantages in improving quality of life over CBIT + PT and PT alone. CBIT might be an appropriate treatment option for patients with tic disorder in Chinese mainland.

## Introduction

Tourette syndrome (TS) and chronic tic disorders (CTD) are neurodevelopmental disorders characterized by the presence of motor tics and/or vocal tics, and at least 1 motor or vocal tic should have lasted for 12 months (1). Typically, tics began at the age of 4–6 years, and the severity of tics is worst between 10 and 12 years and decreases naturally during adolescence and early adulthood (2). Common psychiatric comorbidities among individuals with TS/CTD include obsessive–compulsive disorder (OCD), attention deficit hyperactivity disorder (ADHD), anxiety disorders (AD), and affective disorders (3). Several studies have shown that in patients with TS and CTD, the quality of life (QoL) is impaired, and the presence of comorbidities is correlated with poorer perceived QoL (4, 5). For TS/CTD patients, behavioral therapy (BT) and pharmacotherapy (PT) showed efficacy in reducing tics. In the European clinical guidelines for TS, BT is recommended as the first-line treatment for tic disorders (2, 6).

Comprehensive Behavioral Intervention for Tics (CBIT) as the representative of BT for tic disorders has demonstrated success in reducing tics for children and adults (7–9). In clinical guidelines for the treatment of tic disorders, the American Academy of Neurology (AAN) suggested that CBIT should be offered as an initial treatment option in advance of other psychological interventions and to PT in clinics (10). The core component of CBIT is habit reversal training (HRT), including tic awareness, competing-response training, and social support. Awareness training, includes plenty of techniques that aim to increase awareness of tic happened and associated premonitory urges. Competing response training teaches the patient to do the opposite behavior when they notice the tic has happened or is about to happen. The competing response should apply for 1 min or until the urge to tic disappears and prevents tics from being occurred. Social support involves having a person remind the patient to use the competing response and acclaim the patient for doing the competing response correctly. In addition to HRT, CBIT also includes sessions of psychoeducation, function-based assessment and interventions, relaxation training, and relapse prevention plans (11).

Despite the AAN recommendations, there is still some debate about whether behavioral therapy should be chosen as a first-line option over PT (6), as PT is still generally used as first-line therapy for patients with complex tics (12). PT and BT alone have shown efficacy in tic disorders, e.g., in a recent network meta-analysis including 60 randomized controlled trials (RCTs) found antipsychotic drugs can significantly improve tic symptom score (SMD ranging from −12.32 to −3.20) compared with placebo (13). Another meta-analysis showed BT has a medium effect size (SMD = −0.43, 95% CI: −0.71, −0.16) compared with psychoeducation and supportive therapy (14).

To date, only one study has directly compared PT to behavior therapy in a sample of children and teenagers with CTD and TS, with results showing equal effects in reducing the severity of tic symptoms and improving QoL (15). Given this reality, the newly released European clinical guidelines for TS suggested testing a combination of BT and PT may yield additional treatment benefits (2). No studies have checked out the possible additive effects of combining CBIT and PT as we known.

In Chinese mainland, PT is the first-line treatment for TS or CTD (16), and behavior therapy has low availability. Nevertheless, behavior therapy has shown promise in the Chinese population. One study (17) adopted HRT to 40 children with a tic disorder and found HRT is effective in reducing tics. But no studies have assessed the efficacy of CBIT in Chinese mainland.

The aim of the current study was to investigate the adaptability of CBIT in the Chinese population and evaluate whether CBIT + PT for tics would prove superior to CBIT and PT alone in reducing tics and improving the QoL in a sample of Chinese children and adolescents with TS and CTD.

## Materials and methods

### Study design

This study was conducted in the outpatient clinics in the Department of Psychological Medicine of the Children’s Hospital of Fudan University. Patients were firstly randomized to receive one of two main treatments: CBIT or PT alone. Patients, allocated to CBIT were given the choice to take medicine voluntarily, who decided the choice before any intervention began and knew the grouping scheme at the beginning of the informed consent stage of the project. Thus, it resulted in three groups: CBIT alone (CBIT), CBIT plus pharmacotherapy (CBIT + PT) and pharmacotherapy alone (PT).

The study received approval from the local ethics committee (Approval Number: Children’s Hospital of Fudan University, 2018-290). Before enrollment, a phone screening was conducted, in which the purpose and overall process of the study were described. For eligible participants, a face-to-face visit was held, at which time informed consent/assent was obtained from parents/children, a clinical interview was performed, a baseline assessment was conducted, and randomization occurred.

### Participants

Eligible participants were patients aged 6–16 years with a DSM-5 diagnosis of TS or CTD as given by an experienced child deputy-chief psychiatrist. Inclusion criteria were as follows: Yale Global Tic Severity Scale (YGTSS) > 13 (18). Exclusion criteria included the diagnosis of intellectual disability, learning disorder, and autism spectrum disorder, >4 sessions of habit reversal training or other behavioral treatment for tics, ADHD or OCD diagnoses requiring immediate treatment or change in the current treatment regimen, and unwillingness to participate in the study.

Children who were taking anti-tic medication before enrollment were interviewed, if they are willing to participate in this study. They need to go through a 4 weeks drug washout period before the randomization and the baseline assessment without affecting their condition of tics after the consent of their psychiatrist in charge. Other than the medication prescribed as part of the study, no new medications was started for tics or comorbid conditions during the study.

### Sample size determination and subjects allocation to groups

The sample size in this study was calculated by the GPower3.1 statistical software (19). The main setting parameters were: power = 0.8, alpha = 0.05, effect size *f* = 0.25 (20), symmetry correlation coefficient ρ = 0.5, and non-spherical correction coefficient ε = 1. This study has three treatment groups (CBIT group, PT group, and CBIT + PT group), a total of three assessment levels (baseline, 4 weeks of treatment, and 10 weeks of treatment), using a mixed factors design, the power analysis suggested a sample of 36 patients.

A total of 37 patients were randomly assigned to either CBIT (*n* = 22) or PT (*n* = 15), using a simple randomization plan based on drawing lots in a black box. Patients allocated to the CBIT treatment group were given the choice before CBIT or PT treatment began of whether to also take medicine or only use CBIT. This resulted in a CBIT group (*n* = 12) and a CBIT + PT group (*n* = 10).

During the study, 9/12 participants in the CBIT group completed all sessions of CBIT and 7/10 participants in the CBIT + PT group finished all eight sessions of CBIT.

### Interventions

#### Comprehensive behavioral intervention for tics group

Those receiving CBIT participated in eight sessions over 10 weeks. The first 2 sessions were 90-min long to develop a collaborative relationship and gather information. The remaining sessions were 60-min long. The first 6 sessions happened weekly; the remaining 2, biweekly. CBIT was conducted according to the original English version by Woods et al. (7), which was translated into Chinese version by our group. CBIT was finished by one psychotherapist who is also master degree candidate in clinical psychology and received supervision from a child psychiatrist who was trained at the Queen’s Medical Centre of Nottingham University in the United Kingdom for CBIT training in 2012.

#### Pharmacotherapy group

During the whole study, all randomized patients in this group were assigned to a child/adolescent psychiatrist who was responsible for their medications and related side effect. Thiopride (maximum daily dose not exceeding 600 mg) or aripiprazole (maximum daily dose not exceeding 15 mg) were selected as the medicines for patients. Each subject tried to use a single medicine at a relatively stable dose for 10 weeks of treatment. For patients with very severe symptoms, the medicine reached the maximum daily dose, but the symptoms still seriously affected the patients functioning. In these cases, haloperidol could be used in combination (the maximum daily dose does not exceed 8 mg). If patients experienced extrapyramidal side effects, they took Diphenhydrin Hydrochloride to control side effects.

#### CBIT + PT group

Patients in this group allocated to the CBIT treatment group can further choose whether to take medicine before CBIT treatment began, resulting in CBIT alone and CBIT + PT group. The course of PT and CBIT was as described above.

### Procedures

**FIGURE 1:**
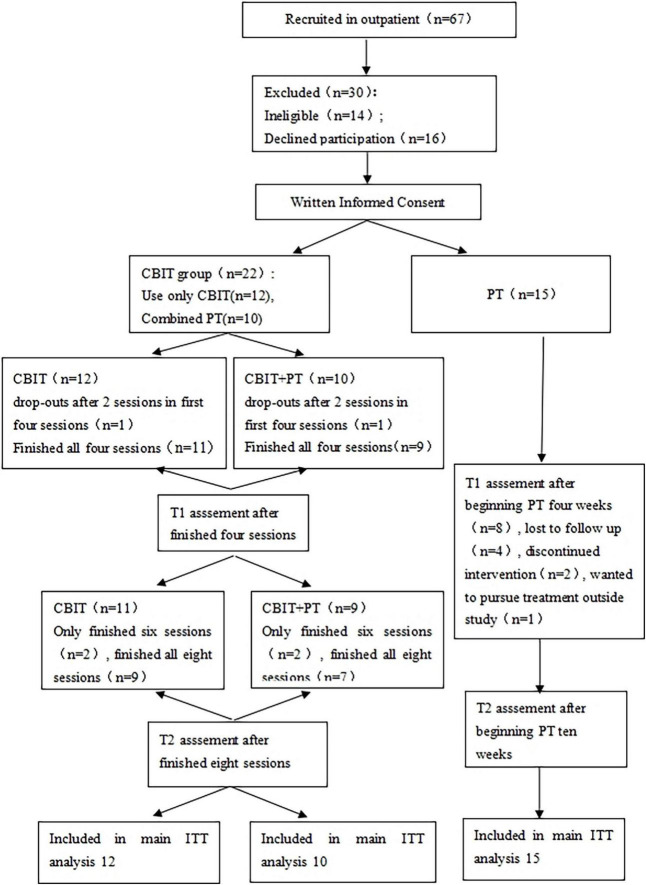
Flow of patients through the trial.

An independent evaluator blind to the treatment assignment of the patients served as the primary assessor in the study. There are three time points to implement assessment for the patients during the study, the first time point is at baseline (T0); the second time point is after the four sessions of CBIT or 4 weeks after beginning PT (T1), and the third time point is performed after the last session of CBIT, or 10 weeks after beginning PT (T2). At each assessment, both the YGTSS and Pediatric Quality of Life Inventory 4.0 version (PedsQL 4.0) of patients were recorded. At T0, the Mini International Neuropsychiatric Interview for Children and Adolescents (MINI-KID) and the parent-reported short version of the SNAP-IV were also administered to assess comorbidity. At T1 and T2, global improvement was assessed using the Clinical Global Impression scale (CGI) for Improvement (CGI-I) (see Figure 1).

### Measures

The MINI-KID is a structured clinical diagnostic interview designed to assess the presence of current psychiatric disorders in children and adolescents aged 6–17 years. The interview was administered to adolescents in the absence of parents (21).

The short version of the Swanson, Nolan, and Pelham Rating Scale (SNAP-IV) Chinese version is a 26-item rating instrument, with each item rated on a 4-point Likert scale from 0 (not at all) to 3 (very much). The core appearance of ADHD, including inattention, hyperactivity/impulsivity, and oppositional symptoms, is rated, respectively; higher scores indicate worse symptoms (22).

The YGTSS is a clinician-rated scale used to assess the severity of tic from two parts, tic symptom, and tic-related impairment. The severity of tic symptoms is assessed separately for motor and a vocal tic on two subscales, and each subscale scores the severity from 0 to 5 on five dimensions, including the number, frequency, duration, intensity, and complexity. The subscales were combined to produce a total tic severity score (ranging from 0 to 50). Lower scores indicate milder tic symptoms, and higher scores indicate severer tic symptoms. Another score ranging from 0 to 50 was assigned for global impairment due to tics, and the lower score indicates less impairment (18).

The Clinical Global Impression scale for Improvement (CGI-I) was used to measure overall treatment response. Improvement is scored from 1 to 7 based on overall clinical changes, in which a score of 1 representing very much improved, 4 representing no change, and 7 indicating very much worse (23).

Pediatric Quality Of Life Inventory (PedsQL™) PedsQL™ has 23 items, scored from 0 (never a problem) to 4 (almost always a problem), which can divide into four subscales: Physical Functioning, Emotional Functioning, Social Functioning, and School Functioning. The Emotional, Social, and School Functioning subscales are combined into a single psycho-social scale. A higher score indicates a higher quality of life. A Total Scale score can also be calculated. The scale includes child self-reports (age range 5–18 years) and parent/carer proxy-reports (age range 2–18 years), and we employed self-report forms and parallel proxy forms (24).

### Statistical analysis

All participants remained in their assigned groups. An intention-to-treat (ITT) analysis was used. All participants assessed at baseline were included, using last-observation carried forward for those lost to post-treatment assessment. For analyses related to non-tic aspects of psychosocial functioning, cases with missing data were excluded on a pairwise basis.

Statistical analyses were performed using the SPSS24.0 software. First, the distribution of quantitative variables was assessed to determine their deviation from the normal distribution within each treatment group (Shapiro–Wilk test) and the homogeneity of variance among the three treatment groups (Levene test). The baseline values of the three treatment groups for each variable were compared using the parametric analysis of variance (ANOVA) test or Pearson’s Chi-Square test with one grouping factor (treatment). The three treatment groups were then compared with regard to change over time for each of the variables using a mixed-model ANOVA with one between-subject factor (treatment) and one within-subject factor (time).

The analysis was repeated including age at the beginning of the intervention as a covariate, to account for differences in age at intervention among groups. This analysis was also repeated including the baseline total score of YGTSS as a covariate to assess the effect of treatment on YGTSS and CGI-I over time, taking into account the tic severity before treatment. The Tukey test was used for multiple comparisons to check whether the absolute scores differed significantly between the time points (T0 vs. T1, T0 vs. T2) for each treatment group, or if the variation in scores differed significantly between treatment groups for each time point. In addition, to evaluate the presence of significant changes in the CGI index as measured by CGI-I score, a one-sample *t*-test with Bonferroni correction was performed for each treatment group, and at every time point assuming a value of 4 as indicative of no change and values significantly lower or higher than 4 as indicating improvement or worsening, respectively, of the CGI over time. All reported *p*-values are two-sided. Statistical significance was set at *p* < 0.05.

## Results

### Baseline

At baseline, no significant difference in demographic and clinical characteristics was found between the three groups (*p* > 0.05) (see Table 1 for details).

**TABLE 1:** The group differences on categorical variables at baseline.

	All subjects (*N* = 37)	CBIT + PT (*N* = 10)	CBIT (*N* = 12)	PT (*N* = 15)	χ^2^/F	*P*
Sex					1.87	0.39
Male	28	6	10	12		
Female	9	4	2	3		
Tic disorder					3.99	0.14
Chronic tic disorder	12	1	6	5		
Tourette syndrome	25	9	6	10		
Comorbidity					1.81	0.41
No	22	5	9	8		
Yes	15	5	3	7		
ADHD	11	3	2	6	1.73	0.42
OCD	4	2	1	1	1.22	0.54
Age						
=9 years	26	10	6	10		
<9 years	11	0	6	5		
M±SD	10.13 ± 2.54	11.3 ± 2.11	9.75 ± 3.12	9.61 ± 2.15	1.54	0.23
YGTSS						
Motor score	12.08 ± 4.86	11.8 ± 3.91	10.67 ± 6.1	13.4 ± 4.24	1.08	0.35
Vocal score	10.19 ± 6.62	12.7 ± 5.27	8.42 ± 6.69	9.93 ± 7.25	1.17	0.32
Severity score	22.27 ± 6.35	24.5 ± 5.7	19.08 ± 4.98	23.33 ± 7.05	2.54	0.09
Impairment score	27.84 ± 7.87	32 ± 6.33	24.17 ± 9	28 ± 6.76	3.01	0.06
SNAP-IV						
Inattention	1.17 ± 0.41	1.19 ± 0.41	1.19 ± 0.31	1.15 ± 0.48	0.03	0.97
Hyperactivity/Impulsivity	1.05 ± 0.47	1.24 ± 0.43	0.91 ± 0.34	1.04 ± 0.55	0.93	0.41
Oppositional defiant	0.94 ± 0.46	0.98 ± 0.54	0.85 ± 0.40	0.98 ± 0.47	0.27	0.77

*P < 0.05, **P < 0.01, ***P < 0.001.

In the dose of the medicines used in the baseline, the difference between CBIT + PT group (364.29 ± 149.20) and PT alone group (416.67 ± 208.17) was not statistically significant [*t*_(23)_ = 0.69, *p* = 0.57].

### Clinical efficacy

#### Outcome indicator 1: Scores of YGTSS

Patients in all three groups showed a significant reduction in tic severity, as assessed by YGTSS severity and impairment scores. For YGTSS severity, the main effect of the group was significant [*F*_(2_,_33)_ = 4.17, *p* = 0.02, η_*p*_^2^ = 0.2], the main effect of time-point was significant [*F*_(2_,_33)_ = 35.05, *p* < 0.001, η_*p*_^2^ = 0.51]. For the Tukey test shown in T1, the CBIT group showed a significantly larger reduction in tic severity than the PT group (*p* = 0.03, 95% CI: 0.52–13.15), no significant interaction was found between time-point and group [*F*_(4_,_33)_ = 0.97, *p* = 0.42].

**FIGURE 2:**
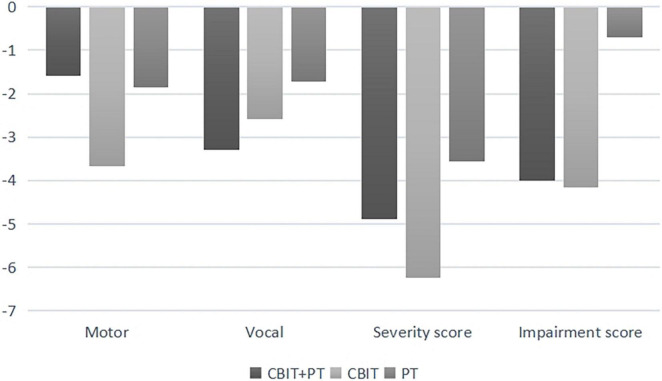
Yale global tic severity scale score variations (means) from baseline (T0) to time 1 (Tl).

**FIGURE 3:**
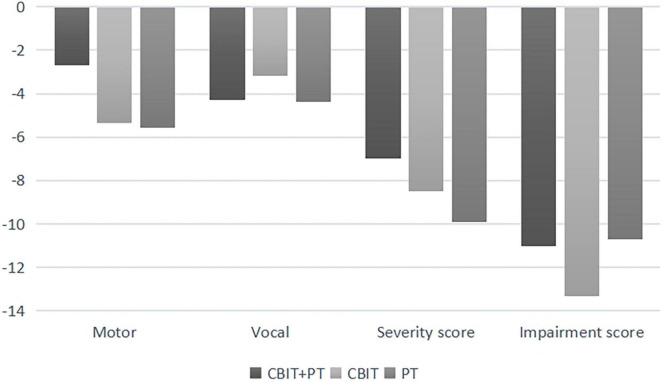
Yale global tic severity scale score variations (means) from baseline (T0) to time 2 (T2).

For YGTSS impairment, the main effect of the group was significant [*F*_(2_,_33)_ = 3.47, *p* = 0.04, η_*p*_^2^ = 0.2], the main effect of time-point was significant [*F*_(2_,_33)_ = 31.71, *p* < 0.001, η_*p*_^2^ = 0.48], no significant interaction was found between time-point and group [*F*_(4_,_33)_ = 0.45, *p* = 0.68] (see Table 2). The variations in YGTSS scores of T1–T0 and T2–T0 are shown in Figures 2, 3; no significant differences were found between the three groups (*p* > 0.05).

**TABLE 2:** Clinical efficacy after different clinical interventions for TS or CTD patients.

		CBIT + PT	CBIT	PT	Treatment effect F (*P*^#^)	Time effect F (*P*^#^)	Treatment × time F (*P*^#^)
							
	Time	M SD	M ± SD	M ± SD			
							
		*n* = 10	*n* = 12	*n* = 15			
YGTSS Motor score	T0	11.80 ± 3.91	10.67 ± 6.10	13.4 ± 4.24	2.87 (*P* = 0.07)	26 (*P* = 0.25)	1.43 (*P* = 0.24)
	T1	10.2 ± 2.94	7 ± 4.13**	11.57 ± 4.9*			
	T2	9.1 ± 3.04	5.33 ± 3.82***	7.86 ± 3.66***			
YGTSS Vocal score	T0	12.7 ± 5.27	8.42 ± 6.69	10.64 ± 6.96	1.08 (*P* = 0.35)	18.6 (*P* = 0.22)	0.6 (*P* = 0.63)
	T1	9.4 ± 6.02**	5.83 ± 5.27*	8.93 ± 6.9			
	T2	8.4 ± 6.15*	5.25 ± 4.9	6.29 ± 6.31**			
YGTSS Severity score	T0	24.5 ± 5.7	19.08 ± 4.98	24.07 ± 6.69	4.17 (*P* = 0.02)*	35.05 (*P<*0.001)***	0.97 (*P* = 0.42)
	T1	19.6 ± 6.96**	12.83 ± 6.21***	20.5 ± 5.68**			
	T2	17.5 ± 7.84*	10.58 ± 5.32**	14.14 ± 7.49***			
YGTSS Impairment score	T0	32 ± 6.33	24.17 ± 9	28.57 ± 6.63	3.47 (*P* = 0.04)*	31.71 (*P<*0.001)***	0.45 (*P* = 0.68)
	T1	28 ± 9.19*	20 ± 8.53**	27.86 ± 6.99			
	T2	21 ± 12.65*	10.83 ± 11.65**	17.86 ± 13.65*			
CGI-I	T0	—	—	—	1.63 (*P* = 0.21)	13.87 (*P* = 0.001)***	1.3 (*P* = 0.29)
	T1	3.1 ± 1.1*	2.58 ± 0.79***	3.43 ± 0.85*			
	T2	2.7 ± 1.34*	2.08 ± 0.9***	2.36 ± 1.22***			

*P < 0.05, **P < 0.01, ***P < 0.001 time 1 or time 2 vs. time 0, within each treatment group.

^#^Greenhouse–Geisser correction for the sphericity assumption.

#### Outcome indicator 2: Score of CGI-I scale

For CGI-I, the main effect of time-point was significant [*F*_(1_,_34)_ = 13.87, *p* = 0.001, η_*p*_^2^ = 0.29], the main effect of group was not significant [*F*_(2_,_34)_ = 1.63, *p* = 0.21], and no significant interaction was found between time-point and group [*F*_(2_,_34)_ = 1.3, *p* = 0.29] (see Table 2). Neither main effect nor interaction effect of time-point and group for clinical efficacy of clinical intervention showed significant when we control the age and baseline total score of YGTSS as covariates (see Supplementary Table 1).

### Quality of life

#### Change of scores of PedQL4.0

The repeated measures ANOVA showed that significant interaction was found between the time-point and group in emotional functioning [*F*_(2_,_29)_ = 4.39, *p* = 0.02, η_*p*_^2^ = 0.23], psychosocial [*F*_(2_,_29)_ = 5.93, *p* = 0.007, η_*p*_^2^ = 0.29], and total QoL score [*F*_(1_,_34)_ = 3.72, *p* = 0.04, η_*p*_^2^ = 0.20] of PedsQL for children suggesting a significantly larger improvement in emotional functioning, psychosocial, and total life quality in the CBIT group for children self-report (see Table 3). The interaction between time-point and group in emotional functioning [*F*_(2_,_26)_ = 5.89, *p* = 0.008, η_*p*_^2^ = 0.31], psychosocial [*F*_(2_,_26)_ = 4.88, *p* = 0.016, η_*p*_^2^ = 0.27] and total QoL score [*F*_(2_,_26)_ = 3.43, *p* = 0.048, η_*p*_^2^ = 0.21] of PedsQL for children were still significant even if we control the age and baseline total score of YGTSS as covariates (see Supplementary Table 2).

**TABLE 3:** The different changes of scores of PedsQL for patients after different interventions.

		CBIT + PT	CBIT	PT	Treatment effect F (*P*^#^)	Time effect F (*P*^#^)	Treatment × time F (*P*^#^)
							
PedQL4.0 for children	Time	M ± SD	M ± SD	M ± SD			
							
		*n* = 9	*n* = 10	*n* = 13			
Physical functioning	T0	80.63 ± 15.72	82.6 ± 25.2	83.85 ± 13.71	0.25 (*P* = 0.78)	0.003 (*P* = 0.96)	1 (*P* = 0.38)
	T2	79.7 ± 11.15	86.81 ± 14.8	82.03 ± 11.7			
Emotional functioning	T0	77.5 ± 18.45	69.44 ± 23.24	74.58 ± 20.94	0.24 (*P* = 0.79)	8.68 (*P* = 0.001)	4.39 (*P* = 0.02)*
	T2	80 ± 15.81	79.44 ± 16.29***	74.17 ± 20.65			
Social functioning	T0	81.5 ± 8.52	88.33 ± 17.32	84.58 ± 19.71	0.24 (*P* = 0.79)	2.4 (*P* = 0.13)	1.84 (*P* = 0.18)
	T2	87.5 ± 8.9	89.44 ± 13.33	85 ± 17.71			
School functioning	T0	71 ± 11.97	84.44 ± 10.74	83.33 ± 13.03	1.35 (*P* = 0.28)	3.86 (*P* = 0.06)	3.22 (*P* = 0.05)
	T2	77 ± 13.38*	86.67 ± 13.46	83.33 ± 13.71			
Psychosocial	T0	76.67 ± 8.85	80.74 ± 11.49	80.83 ± 15.37	0.26 (*P* = 0.77)	15.33 (*P* = 0.001)**	5.93 (*P* = 0.007)**
	T2	81.5 ± 9.8*	85.19 ± 10.59**	80.83 ± 14.95			
Total	T0	78.04 ± 7.17	81.4 ± 14.21	81.88 ± 13.55	0.36 (*P* = 0.56)	4.5 (*P* = 0.04)*	3.72 (*P* = 0.04)*
	T2	80.87 ± 6.57	85.75 ± 9.28**	81.25 ± 13.2			

*P < 0.05, **P < 0.01, ***P < 0.001 time 1 or time 2 vs. time 0, within each treatment group.

^#^Greenhouse–Geisser correction for the sphericity assumption.

The PedsQL for proxy showed a significant main effect of time-point in physical functioning [*F*_(1_,_33)_ = 8.35, *p* = 0.01, η_*p*_^2^ = 0.2], emotional functioning [*F*_(1_,_33)_ = 10.75, *p* = 0.002, η_*p*_^2^ = 0.25], psychosocial [*F*_(1_,_34)_ = 11.38, *p* = 0.002, η_*p*_^2^ = 0.26], and total QoL score [*F*_(1_,_34)_ = 13.21, *p* = 0.001, η_*p*_^2^ = 0.29]; the main effect of group and interaction between time-point and group for sub and the total score was not significant (*p* > 0.05) (see Table 4). The interaction between the time-point and group in emotional functioning [*F*_(2_,_30)_ = 4.08, *p* = 0.03, η_*p*_^2^ = 0.21] and total QoL score [*F*_(2_,_30)_ = 3.51, *p* = 0.04, η_*p*_^2^ = 0.15] of PedsQL for proxy were significant when control the age and baseline total score of YGTSS as covariates, suggesting a significantly larger improvement in emotional functioning and total life quality in the CBIT group for parents reported (see Supplementary Table 3).

**TABLE 4:** The comparisons of the scores of PedsQL for proxy outcome between groups.

		CBIT + PT	CBIT	PT	Treatment effect F (*P*)	Time effect F (*P*^#^)	Treatment × time F (*P*^#^)
							
PedQL4.0 for proxy	Time	M ± SD	M ± SD	M ± SD			
							
		*n* = 10	*n* = 12	*n* = 15			
Physical functioning	T0	81.88 ± 16.06	86.72 ± 15.26	82.14 ± 14.93	0.61 (*P* = 0.55)	8.35 (*P* = 0.01)*	1.22 (*P* = 0.31)
	T2	86.56 ± 11.22	92.45 ± 12.25**	83.48 ± 14.73			
Emotional functioning	T0	71.5 ± 23.34	70 ± 17.19	71.79 ± 18.46	0.087 (*P* = 0.92)	10.75 (*P* = 0.002)	2.92 (*P* = 0.07)
	T2	74 ± 25.25	82.08 ± 14.06***	74.64 ± 19.06			
Social functioning	T0	84.5 ± 14.23	82.5 ± 20.06	79.29 ± 20.83	0.21 (*P* = 0.81)	3.25 (*P* = 0.08)	0.51 (*P* = 0.61)
	T2	85 ± 13.94	85.83 ± 19.29*	81.07 ± 21.14			
School functioning	T0	65 ± 22.49	72.08 ± 22.1	66.07 ± 25.81	0.54 (*P* = 0.59)	3.1 (*P* = 0.09)	0.04 (*P* = 0.96)
	T2	70 ± 16.33	77.08 ± 21.69	66.79 ± 21.72			
Psychosocial	T0	73.67 ± 13.49	75.83 ± 16.32	68.33 ± 22.51	0.87 (*P* = 0.42)	11.38 (*P* = 0.002)**	0.51 (*P* = 0.61)
	T2	78.67 ± 10.77	83.89 ± 17.31*	71.79 ± 22.12			
Total	T0	73.67 ± 13.49	75.83 ± 16.32	68.33 ± 22.51	0.41 (*P* = 0.67)	13.21 (*P* = 0.001)**	1.3 (*P* = 0.29)
	T2	78.67 ± 10.77	83.89 ± 17.31**	71.79 ± 22.12			

*P < 0.05, **P < 0.01, ***P < 0.001, time 2 vs. time 0, within each treatment group.

^#^Greenhouse–Geisser correction for the sphericity assumption.

## Discussion

This study investigated the adaptability of CBIT for a Chinese population and found CBIT was probably effective in reducing tic severity, tic-related impairment, and proved to improve the quality of life in Chinese children and adolescents with TS or CTD. Results also showed that CBIT and PT might be equally effective in reducing tic severity as measured by YGTSS and CGI-I scores. The absolute decrease of 8.5 points (45% from baseline) on the YGTSS severity score in the CBIT group was the same as the decrease of 9.93 points (41%) caused by the effects of medications after 10 weeks of treatment (T2). This is consistent with previous studies on HRT (15), this study reported a decrease in YGTSS severity score of 7.4 points (37%) and 9.41 points (39%) after 10 weeks of treatment with HRT and medicine, respectively.

The current study, to our knowledge, is the first to compare the effectiveness of CBIT + PT combinations with CBIT alone or PT alone in a sample of TS and CTD children and adolescents. It is worth noting that we found CBIT + PT may not be more effective in reducing tic severity than CBIT or PT alone, with the absolute decrease of 7 points (29% from baseline) on the YGTSS severity score in the CBIT + PT group after 10 weeks of treatment. The results are similar to the previous study by O’Connor (25). This study compared the effect of the comprehensive behavioral intervention (CBT) with and without medication in a sample of TS and TD adults and found the scores decrease of 7.72 points (57% from baseline) and 5.58 points (57%) on the Tourette Syndrome Global Scale tics sub-scale after treatment in the CBT + PT group and CBT alone group, respectively, suggesting that equivalent improvement in clinical efficacy. Another study found participants showed tic reduction after CBIT treatment regardless of tic medication status in 9–69 years, including children and adults sample, compared to participants on CBIT + PT (the YGTSS total score decreased by 6.4 points, 25% from baseline), the treatment effect of CBIT alone was significantly larger in participants on no tic medication (the YGTSS total score decrease 7.3 points, 29% from baseline), the results showed PT for TS might not influence the treatment effects of CBIT (26). And in 4 weeks (T1), the CBIT alone group even showed a significantly larger reduction in tic severity than the PT group, it can be explained by the course of PT, at the beginning of PT for tic, the medicine starting from the minimum dose, slowly increase to target treatment dose, the course of PT dependent on the patient’s response to the medication, it may take 4 weeks or more to satisfy the patients, the results in T2 also proved the course of PT.

The present study was designed to investigate the life quality aspects of treatment response. Both CBIT alone and CBIT + PT groups showed improvement in QoL domains, especially in emotional functioning and psycho-social area. The CBIT + PT group also showed significant improvement in school functioning for children self-report. This is possible because both the CBIT and PT can significantly reduce tic severity, and the psychosocial education component in CBIT can reduce parents’ excessive attention to children’s tic symptoms and alleviate the anxiety of parents and children, the social support component and relaxation technique in CBIT can promote the relationship between parents and children, and also improve the child’s emotional regulation skills.

The PT alone group showed no significant improvement in QoL domains. The side effects of PT could account for the results, in the study, 12 of the 15 patients in the PT group reported the side effects of medicines, including drowsiness (7 cases), extrapyramidal side effects (3 cases), weight loss (1 case), and dry mouth (1 case), which may reduce the patient’s quality of life and subjective perception of satisfaction with drug treatment. In summary, our study suggests that compared to PT, CBIT appears to be superior with respect to improve psychosocial functioning and has few side effects. Nevertheless, for achieving higher psychosocial functioning, CBIT might be an effective alternative or adjunct treatment for individuals with TD, especially for those who are worried or afraid of the side effects of PT, or suffer from serious side effects of PT.

Patients in this study also completed a mid-treatment (four sessions using 4 weeks) assessment in addition to assessments conducted post-treatment. The Tukey test showed in 4 weeks, the CBIT group showed a significantly larger reduction in tic severity (the YGTSS severity score decreased 6.4 points, 33% from baseline). This suggests that a shorter course of CBIT (e.g., four individual sessions) can also produce benefits compared to a standard course (eight sessions) of CBIT. For patients who must travel long distances to receive care and for medical settings without enough qualified behavior therapists, reduced sessions might be more convenient and practical and thus patients and their families are likely to be more willing to complete and comply with treatment. Further research on this abbreviated format is warranted. Indeed, a modified four-session CBIT over 3 months study in 2020 has already shown the effects in decreased tic severity in 6–18 years children (the YGTSS severity score decrease by 8.95 points, 46% from baseline) (27), the research suggested that CBIT intervention session might be “dose-specific,” the more sessions the greater improvements.

Our study has a few limitations. First, within the CBIT group, the subjects in the CBIT group and CBIT + PT group were not completely randomized. Although we were testing the feasibility of CBIT, it did alter randomization into the CBIT groups. This likely resulted in baseline YGTSS differences among the groups. Although this difference in baseline severity was not statistically and there were no other statistically significant demographic differences between the groups, it is possible that the groups differed on some other unmeasured factor and that such a factor may have differentially impacted treatment outcomes by condition. Second, to our knowledge, this is the first study of CBIT in Chinese mainland, but our limited sample size limits our ability to confidently generalize our findings to the broader population of China. Future research should replicate and expand upon these findings in large multi-site studies. Finally, the rate of treatment dropouts and those lost to follow-up was high but consistent with a recent meta-analysis of cognitive behavioral therapy dropout, which showed that the rate of the dropout was up to 26.2% during treatment (28). Reasons for these higher non-completion rates are unclear but given that our patients were outpatients, time, and transportation difficulties for patients in other provinces and cities may have been a contributing factor. Likewise for the PT group, the side effects or fear of side effects from PT may have increased the non-completion rate. It suggests a need for preparatory strategies and careful selection and supplementation of treatment setting/delivery in future study.

## Conclusion

This study highlights that CBIT is probably effective in reducing tic severity and improving the quality of life in Chinese children and adolescents with tic disorders. CBIT + PT was possibly not superior to CBIT alone in both reducing tic severity and improvement in QoL. CBIT alone showed advantages in improving quality of life over CBIT + PT and PT alone. CBIT might be provided as a first-line treatment in children and adolescents in Chinese mainland, especially, those patients with mild to moderate tic symptoms if available in the future.

## Data availability statement

The raw data supporting the conclusions of this article will be made available by the authors, without undue reservation.

## Ethics statement

The protocol was approved by the Ethic Committees of the Children’s Hospital of Fudan University (No. 2018-290). Written informed consent to participate in this study was provided by the participants’ legal guardian/next of kin.

## Author contributions

WX contributed to the acquisition of data and drafted the manuscript. QD contributed to the statistical analysis and drafted the manuscript. YZ contributed to revise the manuscript. WJ contributed to the acquisition of data. JH contributed to recruiting the patients. JS made a substantial contribution to the conception of the work and revise the manuscript. All the authors read and approved the final version of the manuscript.
