# Supplementary material for: A preliminary study of comprehensive behavioral intervention for tics in Chinese children with chronic tic disorder or Tourette syndrome
Source: Front Psychiatry. 2022 Nov 11;13:997174. doi: 10.3389/fpsyt.2022.997174 (PMC9691679; doi:10.3389/fpsyt.2022.997174)
Supplement: Supplementary file 1 [file Data_Sheet_1.docx]

Supplementary Material

**Table1** Clinical Efficacy of clinical Intervention

|  | Time | CBIT+PT | CBIT | PT | *Treatment effect F (p^#^)* | *Time effect F (p^#^)* | *Treatment × time F (p^#^)* |
| --- | --- | --- | --- | --- | --- | --- | --- |
|  |  | M±SD | M±SD | M±SD |  |  |  |
|  |  | n=10 | n=12 | n=15 |  |  |  |
| YGTSS  Motor score | T0 | 11.80±3.91 | 10.67±6.10 | 13.4±4.24 | 1.31(*P=*0.28) | 1.41(*P=*0.25) | 1.24(*P=*0.3) |
|  | T1 | 10.2±2.94 | 7±4.13** | 11.57±4.9 |  |  |  |
|  | T2 | 9.1±3.04 | 5.33±3.82** | 7.86±3.66*** |  |  |  |
| YGTSS  Vocal score | T0 | 12.7±5.27 | 8.42±6.69 | 10.64±6.96 | 0.01(*P=*0.98) | 1.56(*P=*0.22) | 0.59(*P=*0.64) |
|  | T1 | 9.4±6.02* | 5.83±5.27 | 8.93±6.9 |  |  |  |
|  | T2 | 8.4±6.15 | 5.25±4.9* | 6.29±6.31** |  |  |  |
| YGTSS  Severity score | T0 | 24.5±5.7 | 19.08±4.98 | 24.07±6.69 | 0.88(*P=*0.42) | 1.06(*P=*0.34) | 1.2(*P=*0.32) |
|  | T1 | 19.6±6.96** | 12.83±6.21*** | 20.5±5.68* |  |  |  |
|  | T2 | 17.5±7.84* | 10.58±5.32*** | 14.14±7.49*** |  |  |  |
| YGTSS  Impairment score | T0 | 32±6.33 | 24.17±9 | 28.57±6.63 | 0.21(*P=*0.81) | 0.31(*P=*0.63) | 0.52(*P=*0.64) |
|  | T1 | 28±9.19 | 20±8.53** | 27.86±6.99 |  |  |  |
|  | T2 | 21±12.65* | 10.83±11.65** | 17.86±13.65* |  |  |  |
| CGI-I | T0 | - | - | - | 0.64(*P=*0.54) | 0.16(*P=*0.69) | 1.75(*P=*0.19) |
|  | T1 | 3.1±1.1* | 2.58±0.79*** | 3.43±0.85* |  |  |  |
|  | T2 | 2.7±1.34* | 2.08±0.9*** | 2.36±1.22*** |  |  |  |

**p<* 0.05, ***p<*0.01,****p<*0.001 time 1 or time 2 vs time 0, within each treatment group.

^#^Covariate = Age and YGTSS total score at the beginning of treatment；Greenhouse–Geisser correction for the sphericity assumption.

**Table 2** Pediatric Quality Of Life Inventory (PedsQL™) for children outcome

| PedQL4.0 for children | Time | CBIT+PT | CBIT | PT | *Treatment effect F (p^#^)* | *Time effect F (p^#^)* | *Treatment × time F (p^#^)* |
| --- | --- | --- | --- | --- | --- | --- | --- |
|  |  | M±SD | M±SD | M±SD |  |  |  |
|  |  | n=9 | n=10 | n=13 |  |  |  |
| Physical  Functioning | T0 | 80.63±15.72 | 82.6±25.2 | 83.85±13.71 | 0.6(*P=*0.56) | 0.67(*P=*0.42) | 0.98(*P=*0.39) |
|  | T2 | 79.7±11.15 | 86.81±14.8 | 82.03±11.7 |  |  |  |
| Emotional  Functioning | T0 | 77.5±18.45 | 69.44±23.24 | 74.58±20.94 | 1.04(*P=*0.37) | 1.45(*P=*0.24) | 5.89(*P=*0.008)** |
|  | T2 | 80±15.81 | 79.44±16.29*** | 74.17±20.65 |  |  |  |
| Social  Functioning | T0 | 81.5±8.52 | 88.33±17.32 | 84.58±19.71 | 0.41(*P=*0.67) | 2.83(*P=*0.09) | 0.72(*P=*0.5) |
|  | T2 | 87.5±8.9 | 89.44±13.33 | 85±17.71 |  |  |  |
| School  Functioning | T0 | 71±11.97 | 84.44±10.74 | 83.33±13.03 | 1.35(*P=*0.28) | 0.08(*P=*0.77) | 1.7(*P=*0.2) |
|  | T2 | 77±13.38* | 86.67±13.46 | 83.33±13.71 |  |  |  |
| Psychosocial | T0 | 76.67±8.85 | 80.74±11.49 | 80.83±15.37 | 0.29(*P=*0.75) | 1.36(*P=*0.25) | 4.88(*P=*0.02)* |
|  | T2 | 81.5±9.8* | 85.19±10.59** | 80.83±14.95 |  |  |  |
| Total | T0 | 78.04±7.17 | 81.4±14.21 | 81.88±13.55 | 0.59(*P=*0.56) | 1.74(*P=*0.20) | 3.43(*P=*0.048)* |
|  | T2 | 80.87±6.57 | 85.75±9.28** | 81.25±13.2 |  |  |  |

**p<*0.05, ***p<*0.01,****p<*0.001 time 1 or time 2 vs time 0, within each treatment group.

^#^Covariate = Age and YGTSS total score at the beginning of treatment；Greenhouse–Geisser correction for the sphericity assumption.

**Table 3** Pediatric Quality Of Life Inventory (PedsQL™) for proxy outcome

| PedQL4.0 for proxy | Time | CBIT+PT | CBIT | PT | *Treatment effect F (p^#^)* | *Time effect F (p^#^)* | *Treatment × time F (p^#^)* |
| --- | --- | --- | --- | --- | --- | --- | --- |
|  |  | M±SD | M±SD | M±SD |  |  |  |
|  |  | n=10 | n=12 | n=15 |  |  |  |
| Physical  Functioning | T0 | 81.88±16.06 | 86.72±15.26 | 82.14±14.93 | 0.75(*P=*0.48) | 1.73(*P=*0.2) | 1.32(*P=*0.28) |
|  | T2 | 86.56±11.22 | 92.45±12.25** | 83.48±14.73 |  |  |  |
| Emotional  Functioning | T0 | 71.5±23.34 | 70±17.19 | 71.79±18.46 | 1.04(*P=*0.37) | 0.07(*P=*0.8) | 4.57(*P=*0.018)* |
|  | T2 | 74±25.25 | 82.08±14.06*** | 74.64±19.06 |  |  |  |
| Social  Functioning | T0 | 84.5±14.23 | 82.5±20.06 | 79.29±20.83 | 0.47(*P=*0.63) | 0.39(*P=*0.54) | 1.06(*P=*0.36) |
|  | T2 | 85±13.94 | 85.83±19.29* | 81.07±21.14 |  |  |  |
| School  Functioning | T0 | 65±22.49 | 72.08±22.1 | 66.07±25.81 | 0.09(*P=*0.91) | 2.39(*P=*0.13) | 0.75(*P=*0.48) |
|  | T2 | 70±16.33 | 77.08±21.69 | 66.79±21.72 |  |  |  |
| Psychosocial | T0 | 73.67±13.49 | 75.83±16.32 | 68.33±22.51 | 0.69(*P=*0.51) | 0.41(*P=*0.53) | 1.52(*P=*0.23) |
|  | T2 | 78.67±10.77 | 83.89±17.31* | 71.79±22.12 |  |  |  |
| Total | T0 | 73.67±13.49 | 75.83±16.32 | 68.33±22.51 | 0.34(*P=*0.71) | 2.6(*P=*0.12) | 3.51(*P=*0.04)* |
|  | T2 | 78.67±10.77 | 83.89±17.31*** | 71.79±22.12 |  |  |  |

**p<*0.05, ***p<*0.01,****p<*0.001, time 2 vs time 0, within each treatment group.

^#^Covariate = Age and YGTSS total score at the beginning of treatment；Greenhouse–Geisser correction for the sphericity assumption.
